# The second violence: Police responses to family violence as a public health exposure

**DOI:** 10.1016/j.puhip.2026.100807

**Published:** 2026-05-08

**Authors:** Kellie McGlynn

**Affiliations:** School of Education, Deakin University, Geelong, VIC, Australia

**Keywords:** Family and domestic violence, Second violence, Policing, Public health, Trauma and violence-informed practice

## Abstract

**Objectives:**

This paper contributes to a broader survivor-led program of research on second violence: the institutional reproduction of harm through systems formally designed to respond to violence. Within that broader program, the paper focuses on one police station encounter to examine how police contact can operate as a modifiable public health exposure in family and domestic violence response.

**Study design:**

Survivor-led autoethnographic case study.

**Methods:**

Data were drawn from *The Second Violence*, a four-year, ethics-approved autoethnographic study documenting lived experience of navigating police and other institutions after separation from an intimate relationship characterised by coercive control and violence. Focusing on a help-seeking episode in which I attended a local police station to report apparent breaches of a Family Violence Intervention Order, I analysed a vignette of this encounter, fieldnotes and analytic memos using feminist autoethnography and Cultural-Historical Activity Theory (CHAT) to examine how objects of work, rules and division of labour shaped safety outcomes.

**Results:**

In this case, police contact operated as a second exposure to harm by intensifying psychological distress through disbelief, minimisation and dismissive institutional responses; delaying safety when patterns of coercive control were not recognised or recorded; leaving risk insufficiently addressed when apparent breaches were not enforced; and eroding trust in systems expected to be protective, with consequences for future help-seeking and engagement with services.

**Conclusions:**

This case suggests that violence prevention and public health responses should address not only perpetrators and primary incidents, but also institutional arrangements that may amplify or attenuate harm. Conceptualising policing as a modifiable public health exposure can inform trauma- and violence-informed practice, integrated risk assessment and survivor-led co-design of responses in which “do no further harm” is treated as a core outcome of family violence systems.

## 1 Introduction

Violence against women and family and domestic violence (FDV) are firmly established as global public health problems [1,2]. Recent World Health Organization (WHO) estimates suggest that almost one in three ever-partnered women worldwide have experienced intimate partner or sexual violence, with little evidence of decline over time [3]. Such exposure is linked to elevated risks of depression, anxiety, post-traumatic stress, suicidality, substance use and multimorbidity across the life course, and is now recognised as a major contributor to women's global burden of disease [4].

In Australia, national surveillance similarly underscores the scale and persistence of FDV: around one in four women and one in 14 men have experienced violence from an intimate partner since the age of 15, and about one in five women have experienced physical and/or sexual partner violence [5]. FDV is a leading contributor to women's burden of disease and accounts for a large proportion of assaults, homicides and sexual offences recorded by police, highlighting its centrality to health and criminal justice systems [5].

From a public health perspective, these figures demonstrate that FDV is not an isolated “private” problem but a population-level determinant of health, shaped by gendered power relations, economic conditions and institutional responses. Public health scholars have long argued that preventing violence requires attention to social determinants and to the systems that respond when harm occurs [1,2,4]. Contemporary reviews of FDV interventions emphasise that health, legal and social care systems can either interrupt trajectories of harm or inadvertently sustain them [[6], [7], [8]].

### 1.1 Conceptualising second violence

In this paper, I conceptualise “second violence” as a form of institutionally mediated harm that occurs when systems designed to recognise, respond to or prevent violence instead reproduce conditions of danger, disbelief or abandonment. The term names a second exposure to harm: not the original violence enacted by the person using violence, but the harm produced through institutional response when help-seeking becomes another site of danger, disbelief or abandonment.

Second violence is therefore not simply an unsatisfactory service encounter, a poor interpersonal interaction or a failure of professional empathy. It is a systemic process through which institutional arrangements can intensify trauma, delay safety, erode trust and extend exposure to risk.

Second violence is related to, but distinct from, secondary victimisation and institutional betrayal [9,10]. Secondary victimisation names the additional harm victim-survivors may experience when institutions respond with disbelief, blame, minimisation or procedural neglect. Institutional betrayal identifies the breach of trust that occurs when an organisation expected to provide protection instead fails to prevent or adequately respond to harm [9,10]. These concepts are central to understanding survivor-system encounters. However, second violence extends this literature by foregrounding institutional response as an exposure in its own right. It asks not only whether a survivor was treated badly or whether an institution failed in its duty of care, but how the organisation of the system itself may produce further violence through its rules, tools, community and divisions of labour.

This conceptualisation is informed by feminist theories of vulnerability and recognisability and by CHAT [[11], [12], [13], [14]]. From this perspective, second violence occurs when victim-survivors’ accounts of danger cannot be made sufficiently recognisable within the activity systems they approach for help. Institutional tools such as forms, counters, scripts, risk assessments, databases and evidentiary categories do not merely record violence; they shape what can be seen, heard and acted upon [13,14]. Rules and routines determine which forms of harm are treated as urgent, credible or actionable. Divisions of labour allocate responsibility for safety, evidence and follow-up, often shifting the burden back onto victim-survivors [13,14]. In this sense, second violence is produced not only by individual disbelief, but by the patterned organisation of institutional activity.

The concept also has a public health dimension. Where harmful institutional responses are repeated across services and jurisdictions, they can become cumulative exposures that contribute to chronic stress, delayed help-seeking, reduced trust in protective systems and prolonged contact with danger [4,[15], [16], [17]]. Second violence therefore links micro-level encounters with macro-level patterns of preventable harm. It offers a way to analyse how systems formally designed to protect may, in practice, contribute to the cumulative health burden of family and domestic violence.

This paper uses one police station encounter to examine second violence in a specific institutional context. However, the concept is intended to have broader analytic reach across survivor-system encounters, including policing, courts, child protection, health, mental health, housing, education and specialist family violence services. Its value lies in making visible the point at which institutional response becomes part of the violence ecology itself: not external to violence, not merely after violence, but implicated in how violence is recognised, prolonged, interrupted or intensified [13,14].

### 1.2 Systems, secondary victimisation and institutional betrayal

The concept of second violence builds on evidence that institutional responses can compound harm for victim-survivors. Research on secondary victimisation and institutional betrayal shows that disbelief, minimisation, blame and inaction by authorities can intensify psychological distress, trauma symptoms, mistrust and reluctance to seek further help [9,10,18]. In family and domestic violence, this matters because institutions such as police, courts, health and social services are not peripheral to safety; they actively shape whether risk is recognised, recorded and interrupted [[6], [7], [8]].

In FDV, policing is a particularly critical institutional site. Internationally and in Australia, public policy encourages victim-survivors to report FDV to police, and many legal protections such as protection orders depend on police enforcement [6,7,18]. Systematic reviews and jurisdictional audits show that police responses can influence the likelihood of further violence, victim satisfaction and wellbeing, and criminal justice outcomes [[6], [7], [8]]. Yet studies also document persistent problems: inconsistent recognition of coercive control, narrow incident-based understandings of risk, variability in recording and enforcing breaches, and organisational cultures that can normalise or minimise FDV, particularly when there are no visible injuries [7,8].

Despite this growing body of work, there remains limited empirical attention to police encounters as health-relevant events: moments in which institutional practice can either alleviate or intensify trauma and risk. Survivor accounts from multiple jurisdictions indicate that routine police contact can be experienced as fragmented, under-resourced and, at times, traumatising, especially where survivors seek protection from patterns of coercive control, digital surveillance, stalking or economic abuse that do not fit incident-based logics [[6], [7], [8]].

However, these accounts are only rarely analysed through a public health lens that conceptualises police contact itself as an exposure with potential cumulative health effects.

### 1.3 Violence as a public health issue and systemic components

In the context of FDV, this research looks beyond perpetrators to understand how police, courts, health and welfare systems co-produce trajectories of harm or safety [1,4,6]. Police contact is one such systemic component. A validating, safety-oriented response at the station or on scene can interrupt escalation, reduce psychological distress and connect survivors with supports [6,7]. A response marked by disbelief, minimisation or procedural deflection can worsen mental health, delay or derail pathways to safety, increase the risk of serious injury or death, and erode trust in systems intended to be protective [4,[8], [9], [10],^16^,^17^].

From a public health standpoint, policing in FDV matters can therefore be understood as a modifiable exposure: a site where organisational objects, rules and routines shape the distribution of risk and protection across populations [7,8,13,14]. Yet most empirical work on policing FDV has been framed in criminological or procedural terms (arrest rates, charging decisions, compliance with policy) rather than in terms of health outcomes, cumulative trauma or population-level risk [[6], [7], [8]].

### 1.4 Survivor-led, systems-informed analysis

Addressing this gap requires methods that can illuminate how institutional arrangements are experienced from the survivor's standpoint while also attending to the systemic dynamics that organise practice. Survivor-led and autoethnographic approaches are increasingly recognised in health and social research as critical for making visible the intersections of structural violence, everyday institutional practice and embodied health consequences [19,20].

This paper draws on The Second Violence: An Autoethnographic Study of Family Violence Services, a four-year, ethics-approved project that examines a survivor-researcher's lived experience of engaging with police, courts, family violence services, legal and mental health providers in Australia [21]. The study combines Butler's feminist theorisation of vulnerability and recognisability [11,12] with CHAT to analyse how tools, rules, community norms and divisions of labour in different institutions shape what happens when someone seeks help [13,14]. Within this broader program of work, the present paper focuses on a single, critical episode of help-seeking: attending a suburban police station to report multiple breaches of a Family Violence Intervention Order after separation from an intimate relationship characterised by coercive control and violence. Through detailed analysis of a narrative vignette, fieldnotes and analytic memos, the paper applies the concept of second violence to one police station encounter.

The aim of this paper is to conceptualise police contact in FDV as a public health exposure and to examine how, in this case, it functioned as a second violence. The study addresses three questions: (1) How were safety, risk and responsibility organised in this episode of police response to FDV? (2) In what ways did police contact operate as a second exposure to harm for a victim-survivor? and (3) What are the implications for public health approaches to violence prevention and system design? This article responds directly to the need for research on systemic components of interpersonal violence, risk and protective factors within systems, and the consequences of system change.

## 2 Methods

### 2.1 Study design

This study uses a survivor-led autoethnographic case study design [19,20] situated within The Second Violence project [21]. Autoethnography uses the researcher's own experience as data to illuminate wider cultural and institutional phenomena [19,20]. A survivor-led approach centres lived experience of violence and system navigation as a primary source of knowledge, aligning with trauma- and violence-informed principles and feminist commitments to making hidden harms visible [19,20,22,23].

The focus on a single, richly documented episode of help-seeking responds to interest in systemic components of interpersonal violence. A detailed case enables close analysis of how institutional practices are organised and experienced at the moment of contact, and how these practices shape trajectories of risk and health [19,20].

### 2.2 Context

The episode analysed occurred in a suburban police station in one Australian state following separation from an intimate relationship characterised by coercive control and multiple forms of abuse. At the time, a Family Violence Intervention Order was in place. The visit to the station formed part of a planned safety strategy and was undertaken to report what were understood as breaches of that order and to seek formal protection.

### 2.3 Case selection

The police station episode was selected from *The Second Violence* project as an information-rich case of survivor-system contact. It was not selected as statistically representative of all police responses to family and domestic violence, but because it offered a bounded and well-documented encounter through which to examine how institutional practices can shape safety, risk and health. The episode was selected for four reasons. First, it involved direct contact with police in relation to an existing Family Violence Intervention Order. Second, it centred on an attempt to report apparent breaches and seek formal protection, making it directly relevant to questions of institutional responsibility and risk recognition. Third, it was documented across several forms of autoethnographic material, including a published vignette, fieldnotes, journal entries and analytic memos. Fourth, it made visible a central contradiction explored in the wider project: the gap between a victim-survivor's attempt to secure safety and an institutional response organised around narrow thresholds of evidence, incident classification and procedural action.

The case was therefore treated as a critical incident within the broader study. Its purpose was to support analytic, rather than statistical, generalisation. The analysis does not claim that all police encounters operate in the same way. Instead, it examines how this encounter reveals institutional mechanisms through which police contact can become a further exposure to harm.

### 2.4 Data sources

Three data sources from *The Second Violence* project were used. First, the literary vignette, The Police Station, reconstructs the visit in first person and was developed from contemporaneous notes and later reflection before being published in de-identified form on the project website [21]. Second, fieldnotes and journal entries recorded before and after the visit documented the sequence of events, observations of the physical environment, bodily and emotional responses, and immediate reflections on the encounter. Third, analytic memos written as part of the broader study identified recurring patterns across system encounters, including disbelief, minimisation, procedural deflection, risk misrecognition and the shifting of responsibility back onto the victim-survivor. Together, these materials provided a layered account of the episode as lived, recorded, reflected upon and theoretically analysed.

### 2.5 Analytic framework and process

Analysis was informed by feminist autoethnography and CHAT [13,14,19,20]. Feminist autoethnography treats personal narrative as embedded in wider structures of gender, power and institutional practice, and is well suited to examining how systems respond to family and domestic violence. CHAT provided a framework for moving from narrative description to analysis of institutional activity [13,14,24,25]. I treated the police station encounter as an activity system and examined how subjects, objects, tools, rules, community norms and divisions of labour shaped what could be recognised, recorded and acted upon.

Analytic work proceeded in four stages. First, I assembled the case corpus, including the vignette, fieldnotes, journal entries and analytic memos relating to the police station encounter. These materials were read chronologically to reconstruct the sequence of the encounter and identify moments where safety, risk, responsibility or institutional action were at stake.

Second, I undertook first-cycle coding close to the narrative material. Initial codes included expressions of fear, uncertainty and bodily distress; police questions and statements; descriptions of the physical environment; moments of minimisation or disbelief; references to evidence, thresholds and breach recognition; and consequences for subsequent help-seeking. This stage preserved the embodied and narrative texture of the autoethnographic material while identifying recurring features of the encounter.

Third, I mapped these codes against the CHAT elements of the activity system [13,14,24,25]. The victim-survivor was analysed as a subject seeking safety, recognition and enforcement. The object of police work was examined through the questions, actions and thresholds that appeared to organise the encounter. The physical setting, including the counter and glass barrier, was analysed as a mediating tool. Incident-based thresholds and evidentiary expectations were analysed as rules. Police occupational norms were considered as part of the community, and the allocation of responsibility for evidence, interpretation and future safety was analysed as division of labour.

Fourth, I synthesised this coding and CHAT mapping into three themes: arriving as an already traumatised body; proceduralised disbelief and the framing of violence as “not a police matter”; and aftermath, delayed safety and erosion of trust. These themes were refined through comparison with the broader study memos and relevant literature on family violence, secondary victimisation, institutional betrayal, policing and public health. The final themes were selected because they captured how the encounter operated not only as an interpersonal interaction, but as a system-organised exposure with implications for safety, health and future help-seeking.

### 2.6 Reflexivity

As both researcher and participant, I occupy a dual position. I write as a white, cisgender woman, a parent, an academic and a survivor of FDV navigating multiple systems. These locations shape what I was able to notice, how I interpreted the encounter and how I was positioned within it. Reflexive journalling and memo-writing were used to attend to the influence of these positions and to situate interpretations within broader scholarship and advocacy around violence and institutional responses.

My experience of second violence also needs to be read through the relative privileges I brought into the police station. I am English-speaking, university educated and familiar with institutional language, documentation and formal complaint processes. These forms of social and cultural capital may have helped me narrate my experience in ways that were more recognisable to police and other services, even when I was still met with disbelief and minimisation. They may also have shielded me from some forms of intensified surveillance, racialised suspicion or exclusion that other victim-survivors encounter.

This matters for the analysis because the harm described in this case should not be read as the most severe possible form of institutional harm. Rather, it illustrates how second violence can occur even for a survivor who holds forms of privilege that may ordinarily increase institutional legibility. For First Nations women, women of colour, migrant and refugee women, women without secure migration status, women with disability, LGBTQIA + victim-survivors, and those experiencing poverty, homelessness or criminalisation, similar institutional practices may be intensified by racism, classed assumptions, heteronormativity, transphobia, language barriers and fears about system intervention. The concept of second violence therefore requires attention not only to gendered harm, but also to the unequal conditions under which victim-survivors become recognisable, credible and worthy of protection.

The use of first person is intentional, consistent with survivor-led autoethnography and with the aim of making system dynamics visible without erasing lived experience [19,20]. At the same time, this is not a claim to represent all victim-survivors. It is a situated account that uses one encounter to examine how institutional arrangements can produce harm, while recognising that those harms are distributed unevenly.

### 2.7 Ethics and safety

*The Second Violence* program of research, including use of autoethnographic data and public vignettes for research, received approval from a university Human Research Ethics Committee. All locations and individuals are de-identified, and legal processes are described in ways that protect privacy while preserving the structural features of the encounter. The project is grounded in trauma- and violence-informed principles: writing and analysis were paced to reduce risk of re-traumatisation, and the public website includes content notes and links to support services [22,23,26,27]. Care was taken to avoid language that shifts responsibility onto victim-survivors or normalises institutional neglect [22,23].

## 3 Results

Three interrelated themes describe how the police station encounter produced harm through institutional response: (1) arriving as an already traumatised body; (2) proceduralised disbelief and the framing of violence as “not a police matter”; and (3) aftermath, delayed safety and erosion of trust. Across these themes, CHAT is used to show how harm was not produced only through an individual officer's response, but through the organisation of the police station as an activity system [13,14]. The analysis attends to the object of work, the tools and physical arrangements that mediated the encounter, the institutional rules through which risk was recognised or dismissed, the community norms shaping police practice, and the division of labour that allocated responsibility for safety, evidence and action.

### 3.1 Arriving already exposed

By the time I drove to the police station, I had experienced prolonged fear, coercive control and cumulative threats. The decision to attend formed part of a carefully planned safety strategy that included filling the car with petrol, packing essential items and preparing for the possibility of not returning home that day. Fieldnotes describe tightness in the chest, difficulty sleeping, intrusive thoughts and a persistent sense of dread. From a public health perspective, this was already a body and family carrying a heavy burden of prior exposure.

Within the police station activity system, I entered as a subject seeking safety, recognition and enforcement of an existing Family Violence Intervention Order. However, the physical and interactional environment mediated the encounter before any formal statement was taken. The waiting area was sparsely furnished, brightly lit and acoustically harsh. The high counter and glass barrier operated as tools of institutional organisation: they managed distance, visibility and access, but also reinforced separation between the person seeking protection and the authority empowered to provide it. These tools did not merely form the background to the encounter; they shaped how safety, vulnerability and credibility could be expressed.

The initial greeting from the officer was brief and informal and was experienced as signalling that my presence was peripheral to the station's immediate priorities. In CHAT terms, this early interaction suggested a tension between two possible objects of work: the victim-survivor's object of securing safety, and the institutional object of managing attendance, workload and procedural thresholds. The vignette captures the dissonance between the gravity of the decision to seek help and the casual tone of the reception [21].

This context matters because police contact does not occur in isolation. It is layered onto existing exposures to violence and stress. Depending on how the activity system is organised, the encounter can function as a point of relief and safety, or as an additional stressor that compounds harm.

### 3.2 Proceduralised disbelief: “not a police matter”

The core of the encounter was shaped by questions and statements that framed my concerns as marginal to police responsibilities. When I explained that there was a protection order and that I believed it had been breached, the officer's questioning focused on whether there had been direct physical contact, whether I had replied to messages, and whether the behaviour met a narrow standard of immediate danger. The officer's focus on discrete physical incidents functioned as an institutional rule within the police station activity system. The repeated attention to direct physical contact, whether I had replied to messages, and whether the behaviour met a threshold of immediate danger narrowed what could be recognised as police-relevant harm. In CHAT terms, this rule shaped the object of work: the encounter became organised around classifying isolated incidents rather than assessing a pattern of coercive control, cumulative breaches and escalating risk [7,8].

This interaction exemplified proceduralised disbelief. Disbelief was not expressed only as overt accusation; it was enacted through the questions asked, the evidence sought, the thresholds invoked and the categories used to sort information. Attempts to explain patterns of coercive control and repeated boundary violations were met with comments that minimised their significance. Phrases suggesting that the situation was “messy” but not criminal, questions about whether I was “sure” I wanted to proceed, and remarks about “victim-centric” services contributing to unrealistic expectations had the effect of undermining my assessment of risk.

The apparent object of police work therefore became the management of reportable incidents rather than the prevention of escalating family violence. The division of labour also shifted responsibility: I was required to make coercive control legible to the institution, while the institution retained authority to decide whether my fear counted as actionable risk.

As a result, patterns of coercive control and cumulative breaches remained largely invisible within police processes. The failure to recognise and record these patterns meant that an opportunity to interrupt the trajectory of violence was missed.

### 3.3 Aftermath: delayed safety and erosion of trust

The consequences of this encounter extended beyond the minutes spent in the station. On leaving, there was no sense of increased safety; instead, there was heightened vulnerability. Fieldnotes describe shaking, difficulty breathing and a sense of exposure and shame. The knowledge that breaches had not been formally recorded or acted upon contributed to a perception that the order was effectively unenforced.

CHAT helps show why this aftermath was not simply an emotional response to an unsatisfactory interaction. It was the consequence of a contradiction within the activity system. The formal object of family violence policing is protection, risk reduction and enforcement of legal orders. Yet in this encounter, the enacted object appeared to be limiting police involvement unless the harm could be organised as a discrete, police-recognisable incident. This contradiction produced a practical safety consequence: the intervention order remained symbolically present but operationally weak.

In the following weeks, the encounter shaped subsequent help-seeking. I became more reluctant to contact police again, even when further incidents occurred. The effort required to recount events, combined with the expectation of disbelief and minimisation, made returning to the station or calling emergency services feel risky in itself. This shifted reliance towards informal strategies and overstretched support services, and contributed to ongoing stress, hypervigilance and sleep disturbance.

The division of labour after the encounter further intensified harm. Responsibility for monitoring risk, interpreting breaches, preserving evidence and deciding whether further contact with police was worth the emotional cost shifted back onto the victim-survivor. Trust in institutions more broadly was also affected. If the agency charged with enforcing protection orders did not view the situation as warranting action, it became difficult to believe that other systems would respond differently.

In this way, police contact operated as a second exposure to harm. It intensified psychological distress, delayed pathways to safety, increased the likelihood that violence would continue and undermined engagement with systems that might otherwise mitigate risk.

## 4 Discussion

This survivor-led case study responds to the framing of FDV as a public health issue by showing how, in one documented encounter, police contact functioned as a form of second violence. The analysis demonstrates that this episode was not only a legal or procedural event; it was also a health-relevant encounter with the capacity to amplify harm. More broadly, the case suggests a mechanism through which institutional responses to family violence may shape health, safety and future help-seeking [4,[8], [9], [10],^16^,^17^].

### 4.1 Violence, systems and cumulative harm

Public health frameworks increasingly recognise that violence is shaped by structural factors and institutional arrangements. This study contributes to that work by articulating “second violence” as a way of understanding harm produced when systems designed to protect victim-survivors instead dismiss or deflect their concerns. Second violence is cumulative: layered on top of interpersonal abuse, it compounds trauma, undermines safety and contributes to the public health burden of violence [4,[15], [16], [17]].

At the individual level, the encounter described here was experienced as acute psychological and embodied distress. At a public health level, the significance of such encounters lies in their cumulative and patterned effects. Repeated institutional responses marked by disbelief, minimisation or inaction can extend exposure to violence, increase anticipatory fear and contribute to chronic stress. Over time, chronic activation of stress responses may contribute to allostatic load, the cumulative physiological burden produced by repeated or prolonged stress exposure [15]. In family violence contexts, this matters because institutional responses that delay safety or discourage future help-seeking may not only worsen immediate distress but also increase the likelihood of ongoing exposure, poorer mental and physical health, and greater service need over time [4,16,17]. The micro-level encounter is therefore health-relevant not because it can be statistically generalised from a single case, but because it illustrates a plausible mechanism through which system contact may contribute to population-level morbidity when repeated across survivors, services and jurisdictions [4,[15], [16], [17]].

The case illustrates a mechanism through which institutional response can become health-relevant: when police contact makes future help-seeking feel unsafe or futile, survivors may delay reporting, rely on informal safety strategies, or remain exposed to escalating violence for longer. Across a population, repeated patterns of delayed protection, chronic stress and reduced trust in services may contribute to preventable morbidity, including trauma symptoms, sleep disturbance, anxiety, depression, multimorbidity and injury, as well as increased risk of serious harm or death [4,5,8,[15], [16], [17]].

### 4.2 Policing as a modifiable public health exposure

Conceptualising policing as a public health exposure highlights that institutional practices are not fixed; they can be reshaped through policy, training, resourcing and accountability [[6], [7], [8]]. Exposure includes not only contact with individual officers but also the organisational objects, rules and divisions of labour that structure their work [13,14].

If the primary object of police work in family violence is understood as managing discrete incidents within limited thresholds, institutional arrangements will continue to miss patterns of coercive control and fail to enforce protection orders [7,8]. If, instead, the object is reframed as preventing harm and supporting safety, then tools, protocols and performance indicators can be aligned with that aim. This might include:

• trauma- and violence-informed approaches to first contact, including validation, clear explanation of options and efforts to minimise further harm [22,23,26,27].
• risk assessment processes that incorporate patterns of behaviour and cumulative breaches, not just single incidents
• integrated information systems that ensure breaches are recorded and visible across agencies
• supervision and accountability mechanisms that support active enforcement of orders and responsiveness to victim-survivors’ assessments of risk

Such changes would position police stations as critical sites for interrupting, rather than reproducing, trajectories of violence.

### 4.3 Survivor-led evidence for system redesign

The study also underscores the value of survivor-led research for informing public health responses [19,20]. Administrative data provides essential information about prevalence, risk factors and outcomes, but they cannot show how institutional arrangements are experienced or how they produce second violence. Survivor narratives, when analysed systematically, offer evidence about where systems fail, where they succeed and how they might be redesigned [19,20]. By presenting this case both on a public website and in a peer-reviewed format, *The Second Violence* seeks to bridge public and professional conversations and position survivor insight as crucial data on system behaviour [21].

### 4.4 Strengths and limitations

A strength of this study is its fine-grained, situated account of a single system encounter, analysed through theoretical and experiential lenses. The survivor-led design centres the perspectives and priorities of a victim-survivor, and the use of CHAT supports attention to how organisational structures and objects shape practice [13,14,19,20].

Findings are not statistically generalisable and do not represent all police responses. The claims made here are deliberately bounded: the case demonstrates how one police encounter was experienced as institutionally mediated harm. It does not establish the prevalence of such encounters, nor the extent to which the same mechanisms operate across all policing contexts. However, when read alongside existing evidence on family violence policing, secondary victimisation and institutional betrayal, the case suggests mechanisms that may have broader analytic resonance: incident-based risk recognition, evidentiary thresholds, procedural deflection and the shifting of responsibility back onto victim-survivors [[7], [8], [9], [10],^18^].

The concept of second violence may be particularly transferable to survivor-system encounters where people seek recognition, protection or care from institutions that hold authority over safety, credibility or access to support. These include encounters with police, courts, child protection, health and mental health services, housing systems, education settings and specialist family violence services. In each context, the concept directs attention to how institutional tools, rules, community and divisions of labour may either interrupt harm or reproduce it [13,14]. Transferability therefore rests not on similarity of setting alone, but on the presence of comparable institutional dynamics: help-seeking under conditions of vulnerability, unequal power between survivor and system, contested credibility, and the possibility that procedural responses may intensify rather than reduce harm.

The dual role of the author as survivor and researcher may introduce bias, but it also enables access to data that would otherwise be difficult to obtain. Reflexive practice and engagement with existing literature were used to situate and check interpretations.

### 4.5 Implications for public health practice and policy

The implications below should be read as arising from the case in dialogue with the wider literature on family violence, policing, secondary victimisation and public health, rather than as direct claims from a single encounter alone [4,[6], [7], [8], [9], [10],[16], [17], [18]]. The case provides an analytic example of how institutional arrangements can produce harm; the broader implications identify areas where this mechanism may warrant further empirical investigation, policy review and survivor-led system redesign.

For public health practitioners and policymakers, several implications follow from reading this case alongside existing evidence:

1. Recognise police contact as a public health event in family and domestic violence, with potential to reduce or increase harm.
2. Embed trauma- and violence-informed principles into policing policy, training and supervision, with a focus on “do no further harm” [22,23,26,27].
3. Design risk assessment and recording systems that capture patterns of coercive control and cumulative breaches, not only discrete incidents [7,8].
4. Incorporate measures of procedural justice, trust and safety into performance indicators for family violence response [[6], [7], [8],^18^].
5. Support survivor-led co-design and review of family violence pathways, ensuring that lived experience informs system priorities and practice [19,20].

Addressing these issues would align policing more closely with public health goals of reducing population-level harm from violence.

## Ethical statement

*The Second Violence* project, including use of autoethnographic data and public vignettes for research, was approved by Deakin University Human Research Ethics Committee (2025/HE000632).

## Funding

This research received no specific grant from any funding agency in the public, commercial or not-for-profit sectors.

## Declaration of competing interest

The author declares no competing interests or conflicts of interest.
